# Infections in the Era of Targeted Therapies: Mapping the Road Ahead

**DOI:** 10.3389/fmed.2020.00336

**Published:** 2020-08-18

**Authors:** Leonard H. Calabrese, Cassandra Calabrese, Tiphaine Lenfant, Elizabeth Kirchner, Vibeke Strand

**Affiliations:** ^1^Rheumatic & Immunologic Disease, Cleveland Clinic, Cleveland, OH, United States; ^2^Assistance Publique des Hôpitaux de Paris, Université de Paris, Paris, France; ^3^Hôpital Européen Georges Pompidou, Service de Médecine Interne, Paris, France; ^4^Division of Immunology and Rheumatology, Stanford University, Palo Alto, CA, United States

**Keywords:** targeted therapies, TNF inhibitors, infection, tuberculosis, natalizumab, PML, vaccine

## Abstract

Immunosuppressive treatment strategies for autoimmune diseases have changed drastically with the development of targeted therapies. While targeted therapies have changed the way we manage immune mediated diseases, their use has been attended by a variety of infectious complications—some expected, others unexpected. This perspective examines lessons learned from the use of different targeted therapies over the past several decades, and reviews existing strategies to minimize infectious risk. Several of these infectious complications were predictable in the light of preclinical models and early clinical trials (i.e., tuberculosis and TNF inhibitors; meningococcus; and eculizumab). While these scenarios can potentially help us in terms of enhancing our predictive powers (higher vigilance, earlier detection, and risk mitigation), targeted therapies have also revealed unpredictable toxicities (i.e., natalizumab and progressive multifocal leukoencephalopathy). Severe infectious complications, even if rare, can derail a promising therapeutic and highlight the need for increased awareness and meticulous adjudication. Tools are available to help mitigate infectious risks. The first step is to ensure that infection safety is adequately studied at every level of drug development prior to regulatory approval, with adequate post-marketing surveillance including registries that collect real-world adverse events in a collaborative effort. The second step is to identify high risk patients (using risk calculators such as the RABBIT risk score; big data analyses; artificial intelligence). Finally, the most underutilized interventions to prevent severe infections in patients receiving targeted therapies across the spectrum of immune mediated inflammatory diseases are vaccinations.

## Introduction

Immunosuppressive treatment strategies for autoimmune diseases have changed drastically over the past 25 years. There has been a shift from broad and relatively non-specific agents such as glucocorticoids, antimetabolites and alkylators, to an increasing array of therapies targeting discrete molecular structures within the immune system. These include biologic therapeutics directed at soluble effectors such as cytokines, immunoglobulins, and complement as well as cellular targets. More recently introduced agents include an expanding number of small molecules directed at a number of intracellular signaling pathways. Finally, increasingly complex therapeutic strategies are in development, including cellular therapies with bioengineered receptors directed at autoreactive cells or tolerogenic strategies employing cytokines or bioengineered cells with endogenous inhibitory capacity (1). Increasingly these therapies will be utilized in a variety of combinations. The capacity for immune modulation may be inexhaustible.

Attendant to this revolution in immune based therapies, aside from their variable but often remarkable efficacy, are a spectrum of new toxicities. Most prominent among these are serious and opportunistic infections. It is clearly beyond the scope of this review to discuss the spectrum of infections associated with targeted therapies; however, the European Society of Clinical Microbiology and Infectious Diseases has recently published a series of papers reviewing the infection safety profile of a majority of currently employed targeted therapies for immune based diseases (2–6). From these data it can be readily appreciated that infectious complications not only represent a serious source of morbidity and mortality but, in our opinion, should be considered the Achilles heel of the class. As we move ahead to even more potent and complex therapeutic strategies, we should pause to consider the lessons we've learned from the first two decades of targeted therapies and strategies we can employ now to minimize infectious risks moving forward.

## Perspective

### Lessons Predictable and Unpredictable

Though imperfect, tools are available for predicting infections associated with immune based therapeutics. One preclinical source of translational insight are genetically manipulated mice with humanized immune systems utilized to study innate and adaptive immune responses to infectious diseases in an effort to align biology with expected clinical outcomes (7). Of even greater relevance may be our growing knowledge of over 250 monogenic primary immune deficiency states where disruption of immune related molecular pathways leads to serious or opportunistic infections as part of their phenotypes (8). Questions now include what have we learned from these models and long-term clinical experience and how should we exploit them to our advantage?

The first major therapeutic class introduced were biologic agents inhibiting TNF (TNFi); infliximab and etanercept were approved in 1998, followed by adalimumab (2002), certolizumab (2008), and golimumab (2009) (6). Upon approval no risk mitigation strategy was mandated regarding tuberculosis (Tb) as no signal had emerged in the relatively small pivotal trials conducted at the time. In retrospect this low level of concern for Tb may seem curious in light of the fact that several years prior preclinical models had demonstrated that TNF was essential for integrated host defenses against Tb (9) and other opportunistic infections (OI). By 2001, initial reports in the literature of Tb infection in the setting of infliximab therapy (10) were followed by increasing reports of atypical mycobacterial infections with etanercept and multiple Tb and OI adverse events in the clinical development program for adalimumab (11, 12). Following approval of adalimumab in December 2002, the FDA held an Arthritis Advisory Committee meeting in March 2003 specifically to discuss adverse events associated with use of TNFi, including serious and opportunistic infections and malignancies (11). Following this recognition, in addition to the required post-marketing surveillance programs already underway, more detailed risk mitigation was introduced and currently Tb reactivation is a diminishing issue for patients receiving TNFi in Western (i.e., low incidence) countries, although risks persist in areas of the world with higher baseline Tb prevalence (13). The scenario of Tb with TNF inhibitors should be considered a missed opportunity, as foreknowledge of potential Tb reactivation could have led to heightened vigilance and potentially earlier detection and risk mitigation.

A second example of an infection risk predicated from primary immunodeficiency states where aggressive risk mitigation was deployed stems from the association of late acting complement component deficiencies, Neisserial infections, and the therapeutic agent eculizumab (14). Eculizumab is a monoclonal antibody directed against the complement component C5. It was approved by the FDA in 2007 to treat atypical hemolytic uremic syndrome and in 2011 for paroxysmal nocturnal hemoglobinuria (PNH). At the time of its development it was well-known that the terminal components of the complement pathway played an important role in innate immune defenses against invasive meningococcal disease. Based on work in animal models as well as observations in primary immunodeficiency states, it was believed that the terminal sequence of complement was critical for host defense against invasive and recurrent meningococcal infections (15, 16). Subsequently it is now known that eculizumab is associated with a 1,000–2,000 times greater risk of Neisseria infections (14). Based on this modeling, all patients in the small phase 2 pilot randomized controlled trial (RCT) were immunized for Neisseria and no infections occurred. Despite this risk mitigation strategy, 2 of 196 PNH patients in the pivotal trials developed meningococcal sepsis and a boxed warning was added upon approval (https://www.accessdata.fda.gov/drugsatfda_docs/label/2007/125166lbl.pdf) (17). Data have shown that patients may develop meningococcal disease even after receiving appropriate vaccinations, as the majority of eculizumab-associated meningococcal infections have been non-groupable Neisseria meningitides (14). Current recommendations call for the administration of both the meningococcal conjugate and serogroup B meningococcal vaccines at least 2 weeks prior to administering the first dose of eculizumab (14). Despite these efforts, breakthrough infections have been reported and antibiotic prophylaxis is required through vaccine series completion (18). Development and approval of eculizumab offers an example of planned risk mitigation based on preclinical and clinical data.

While these tools can potentially serve us well in terms of enhancing our predictive powers, targeted therapies have also revealed surprises. The most notable example of unexpected toxicity with far-ranging implications in association with a targeted therapy can be dated to February 28, 2005, when natalizumab, a promising anti-integrin monoclonal antibody for treatment of multiple sclerosis (MS) was withdrawn from RCTs because 3 patients (two with MS and one with Crohn's disease) developed progressive multifocal leukoencephalopathy (PML), a rare and frequently fatal demyelinated central nervous system infection caused by reactivation of the John Cunningham virus (19). PML is a rare demyelinating disease previously reported predominantly in the setting of severe immunosuppression from cancer, transplantation and especially active HIV (20). Neither preclinical models nor primary immunodeficiency diseases linked to integrin-targeted pathways had revealed hints of PML (although this is an infection where animal models are insufficient) making this association even more striking. Another biologic agent, efalizumab, a targeted therapy directed against lymphocyte function-associated antigen-1 (LFA-1) indicated for treatment of psoriasis, was linked to several cases of PML and subsequently voluntarily removed from the market (19). Over time, natalizumab has been reintroduced as a treatment for both MS and Crohn's disease under a rigorous risk management program (21). Although previously no rheumatic disease patient receiving a biologic agent had been diagnosed with PML, in 2006 two cases were reported in patients treated with rituximab for SLE (22). Prior to this, PML had been reported in association with rituximab use only in the setting of hematologic cancers. As a result of these reports the pale of PML was felt across multiple specialties (neurology, dermatology, rheumatology, and gastroenterology) and it took many years to develop data-driven recommendations to minimize these risks (20).

Lessons learned from these vignettes should be that severe infectious complications, even if rare, can derail a promising therapeutic, and that awareness of and meticulous adjudication for even theoretically possible complications is critical to the clinical development program, approval process and post-marketing surveillance.

Finally, an emerging class of drugs that inhibit kinases of the Janus family (JAK) hold valuable lessons in predicting and managing infection-related safety. Janus kinases play critical non-redundant roles in mediating cellular transcriptional responses to cytokines, and in cell activation, survival, and proliferation. They are now approved for use in a growing list of immune mediated diseases (23). Lessons from patients with primary immunodeficiency states reveal that defects, particularly in JAK3, are well known to be associated with severe combined immunodeficiency syndrome, and support a critical role of JAKs in defense against serious and opportunistic infections (24). A particular class effect of JAK inhibitors is an increased risk of herpes zoster (HZ) (25). The baseline risk of HZ in rheumatoid arthritis is 1.5–2-fold higher compared to the general population, and the incidence of HZ reported with tofacitinib, which preferentially inhibits JAK1, JAK2, and JAK3, is double that reported in RA (25). A similar degree of risk of HZ is seen with baricitinib, a JAK1 and 2 inhibitor, and upadacitinib, a JAK1 inhibitor which have been more recently approved (Winthrop) and thus it appears to be a class effect. Despite this increased risk, HZ in this setting is rarely multidermatomal or disseminated. For reasons that remain unknown, the rates of HZ with tofacitinib in Japan and Korea are reported to be nearly 2–3 times higher than those observed in the United States or Europe (26). Based on these data there is a strong rationale to vaccinate against HZ with the recombinant subunit vaccine in all patients before they begin therapy (27).

**Figure 1:**
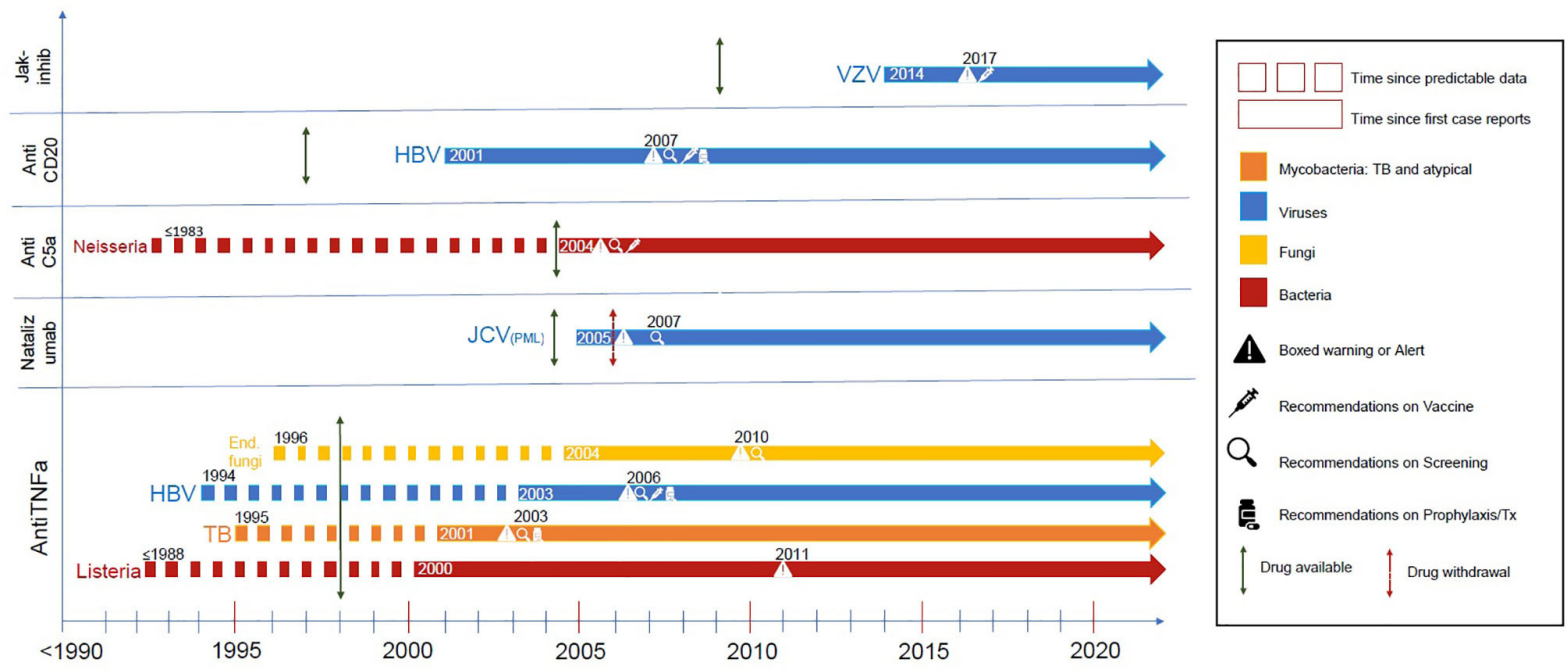
These are representative infectious complications from major targeted therapies. The dotted line demonstrates the time where there was supportive evidence either from preclinical models, clinical case reports, or evidence from primary immunodeficiency states was apparent. The dotted line becomes solid at about the time of the first case reports of this complication, followed by a green arrow indicating date of drug approval and a red arrow indicating drug withdrawal if such occurred. Additional symbols (see legend) indicate regulatory modifications of labeling or guidelines of care (screening, vaccination, or prophylaxis) when they occurred. References are in the supplement (Supplemental Data 1).

Figure 1 displays a chronologic history of key targeted therapies with the biologic background relevant to predicting infectious complications with subsequent dates of official reporting.

### Reducing Risks of Serious and Opportunistic Infections

#### Monitoring and Data Collection

The first step in reducing risk of serious and opportunistic infections is to assure that infection safety is adequately studied at every level of drug development prior to and after regulatory approval. This process has improved, as evidenced by the increase in the size and duration of RCTs for regulatory approval in clinical development programs. The first biologics (e.g., etanercept) involved fewer than 1,000 patients in their pivotal clinical development programs. By 2002 the development program for adalimumab exceeded 2,500 patient years; more recently approved synthetic targeted therapies such as tofacitinib and upadacitinib were tested in 4,816 and 4,443 patients, respectively, representing 5,716 (28) and 5,106 (29) patient years of exposure. Despite the increase in the number of subjects treated, it remains challenging to efficiently detect opportunistic infections which have an incidence ranging from <1 to ~5/1,000 patient years (30, 31) and 0.02 events/1,000 patient years for PML associated with rituximab therapy for rheumatoid arthritis (RA) (32).

It is well-known that RCT data are limited in their relevance, as the studied populations are under-representative of comorbidities present in the general population, thereby increasing the likelihood for infectious complications. Registries are powerful tools to study rare real-world adverse events (33). Unfortunately, they are not homogeneous in terms of patient selection and populations making straightforward comparisons difficult. Efforts to enhance the utility of registries which have borne success include collaborative efforts to create case definitions (31) and informatic approaches to harmonize registry data (34).

#### Biomarkers to Predict Serious Infections

Identifying high risk patients is an important goal of mitigating infectious risk. Multiple approaches have been advocated with several tools currently available or in development. Clinical algorithms can identify high-risk clinical scenarios, and some have been validated including one derived from the German biologic RABBIT registry (35). This RABBIT risk score estimates the probability of an RA patient experiencing a serious infection during the next 12 months, based on data from patients with similar risk profiles. This instrument can be freely accessed on the internet (https://biologika-register.de/en/rabbit/rabbit-risk-score-of-infections/) and demonstrates that risks resulting from higher age, poor functional status, co-morbidities and treatment are interrelated. For example, when applying the risk calculator, a 65-year-old RA patient with low disease activity on prednisone 5 mg has a predicted serious infection risk of 2.4% with TNF inhibition. However, if prednisone usage is 15 mg daily or higher, this risk increases to 9%. The search for a laboratory-based biomarker to identify high risks for infectious complications is also advancing. One example, the multiple biomarker disease activity (MBDA) test, is a validated 12-protein biomarker assay used to monitor RA disease activity. Correlating MBDA with hospitalizations for serious infections linked to the Medicare database revealed significant risk (HR 1.32 CI 1.23–1.41) controlling for potential confounders (36). Further work in this area incorporating artificial intelligence and big data is awaited.

#### Vaccines to Prevent Infections

Clearly the greatest and most underutilized intervention to prevent infections in patients receiving targeted therapies across the spectrum of immune mediated inflammatory diseases is vaccination. Patients with immune mediated diseases are more vulnerable to acquisition of vaccine preventable infections as well as more likely to suffer greater morbidities and mortality (37, 38). Although concerns that efficacy, immunogenicity, and safety may be adversely affected by such therapies, limited evidence indicates that benefits outweigh risks and a growing body of data suggests that such patients, when vaccinated, develop appropriate immunogenic responses when receiving most but not all targeted therapies (39). In 2013, the Infectious Diseases Society of America published a position statement regarding vaccinating the immunosuppressed host, and clearly asserted that specialists who care for immunocompromised patients share responsibility with primary care providers for ensuring that appropriate vaccinations are administered (40). Accordingly, many professional organizations have guidelines or recommendations concerning vaccination of patients with the diseases of interest. Perhaps the most thorough guidance document comes from the European League Against Rheumatism's (EULAR) updated set of recommendations for vaccinating adult patients with inflammatory diseases (41), accompanied by a systematic literature review regarding efficacy, immunogenicity, and safety of vaccines in this population (39). Efforts such as these have set the bar for strong and evidence-based recommendations for clinicians.

Despite evidence that vaccines are safe and efficacious in the immune mediated inflammatory disease population, there remains a uniform suboptimal uptake of vaccinations in patients with immune mediated rheumatic, dermatologic, and gastrointestinal diseases (42–47). Reasons for this are multifold but lack of provider recommendations (48) and poorly designed or absent system designs are prominent reasons (49–51). This is an area of active research and strategies to overcome this have been successfully adopted—the most successful using a combination of interventions (50, 51). Finally, and unfortunately, resistance to vaccination is also a growing threat not only to patients with immune mediated diseases receiving targeted therapies but also the public at large; the World Health Organization has named vaccine hesitancy, a “reluctance or refusal to vaccinate despite the availability of vaccines,” among the top 10 threats to health worldwide (52).

## Data Availability Statement

The original contributions presented in the study are included in the article/Supplementary Material, further inquiries can be directed to the corresponding author.

## Author Contributions

LC wrote the first draft of the manuscript. LC, CC, VS, and EK wrote sections of the manuscript. TL and CC designed the figure. All authors contributed to manuscript revisions, reviewed, and approved the submitted version.

## Conflict of Interest

The authors declare that the research was conducted in the absence of any commercial or financial relationships that could be construed as a potential conflict of interest.
