# Supplementary material for: Infections in the Era of Targeted Therapies: Mapping the Road Ahead
Source: Front Med (Lausanne). 2020 Aug 18;7:336. doi: 10.3389/fmed.2020.00336 (PMC7461856; doi:10.3389/fmed.2020.00336)
Supplement: Supplementary file 1 [file Data_Sheet_1.docx]

Supplementary Material

# 1 Supplemental references for Figure 1

**1.1 TNF inhibitors**

**1.1.1 Listeria monocytogenes**

1. Nakane A, Minagawa T, Kato K. Endogenous tumor necrosis factor (cachetin) is essential to host resistance against Listeria monocytogenes infection. Infect. Immun. 1988;56:2563–9.
2. Morelli J, Wilson FA. Does administration of infliximab increase susceptibility to listeriosis? Am. J. Gastroenterol. 2000;95:841–2.
3. Slifman NR, Gershon SK, Lee J-H, Edwards ET, Braun MM. Listeria monocytogenes infection as a complication of treatment with tumor necrosis factor alpha-neutralizing agents. Arthritis Rheum. [Internet] 2003 1;48:319–24
4. <https://www.fda.gov/drugs/drug-safety-and-availability/fda-drug-safety-communication-drug-labels-tumor-necrosis-factor-alpha-tnfa-blockers-now-include>

**1.1.2 Mycobacterium tuberculosis**

1. Flynn JAL, Goldstein MM, Chan J, Triebold KJ, Pfeffer K, Lowenstein CJ, et al. Tumor necrosis factor-α is required in the protective immune response against mycobacterium tuberculosis in mice. Immunity 1995;2:561–72.
2. Núñez Martínez O, Ripoll Noiseux C, Carneros Martín JA, González Lara V, Gregorio Marañón HG. Reactivation tuberculosis in a patient with anti-TNF-alpha treatment. Am. J. Gastroenterol. 2001;96:1665–6.
3. Gardam MA, Keystone EC, Menzies R, Manners S, Skamene E, Long R, et al. Anti-tumour necrosis factor agents and tuberculosis risk: Mechanisms of action and clinical management. Lancet Infect. Dis. 2003 1;3:148–55.

**1.1.3 HBV**

1. Guidotti LG, Ando K, Hobbs M V, Ishikawa T, Runkel L, Schreiber RD, et al. Cytotoxic T lymphocytes inhibit hepatitis B virus gene expression by a noncytolytic mechanism in transgenic mice. Proc. Natl. Acad. Sci. U.S.A. 1994;91:3764–8.
2. Michel M, Duvoux C, Hezode C, Cherqui D. Fulminant hepatitis after infliximab in a patient with hepatitis B virus treated for an adult onset still’s disease. J. Rheumatol. 2003;30:1624–5.
3. Nathan DM, Angus PW, Gibson PR. Hepatitis B and C virus infections and anti-tumor necrosis factor-α therapy: Guidelines for clinical approach. J. Gastroenterol. Hepatol. 2006;21:1366–71.

**1.1.4 Endemic fungi**

1. Huffnagle GB, Toews GB, Burdick MD, Boyd MB, McAllister KS, McDonald RA, et al. Afferent phase production of TNF-alpha is required for the development of protective T cell immunity to Cryptococcus neoformans. J. Immunol. 1996;157.
2. Allendoerfer R, Deepe GS. Blockade of endogenous TNF-alpha exacerbates primary and secondary pulmonary histoplasmosis by differential mechanisms. J. Immunol. 1998 15;160:6072–82.
3. Bergstrom L, Yocum DE, Ampel NM, Villanueva I, Lisse J, Gluck O, et al. Increased risk of coccidioidomycosis in patients treated with tumor necrosis factor α antagonists. Arthritis Rheum. 2004;50:1959–66.
4. Wallis RS, Broder MS, Wong JY, Hanson ME, Beenhouwer DO. Granulomatous infectious diseases associated with tumor necrosis factor antagonists. Clin. Infect. Dis. 2004 1;38:1261–5.
5. Hage CA, Bowyer S, Tarvin SE, Helper D, Kleiman MB, Wheat LJ. Recognition, diagnosis, and treatment of histoplasmosis complicating tumor necrosis factor blocker therapy. Clin. Infect. Dis. 2010 1;50:85–92.

**1.2 Natalizumab (JCV)**

1. Langer-Gould A, Atlas SW, Green AJ, Bollen AW, Pelletier D. Progressive multifocal leukoencephalopathy in a patient treated with natalizumab. N. Engl. J. Med. 2005 28;353:375–81.
2. Kappos L, Bates D, Hartung H-P, Havrdova E, Miller D, Polman CH, et al. Natalizumab treatment for multiple sclerosis: recommendations for patient selection and monitoring. Lancet. Neurol. 2007;6:431–41.

**1.3 Anti C5a (Neisseria meningitidis)**

1. Ellison RT, Kohler PF, Curd JG, Judson FN, Reller LB. Prevalence of congenital or acquired complement deficiency in patients with sporadic meningococcal disease. N. Engl. J. Med. 1983 21;308:913–6.
2. Hillmen P, Hall C, Marsh JCW, Elebute M, Bombara MP, Petro BE, et al. Effect of Eculizumab on Hemolysis and Transfusion Requirements in Patients with Paroxysmal Nocturnal Hemoglobinuria. N. Engl. J. Med. 2004 5;350:552–9.
3. Rother RP, Rollins SA, Mojcik CF, Brodsky RA, Bell L. Discovery and development of the complement inhibitor eculizumab for the treatment of paroxysmal nocturnal hemoglobinuria. Nat. Biotechnol. 2007;25:1256–64.

**1.4 Anti CD20 (HBV)**

1. Dervite I, Hober D, Morel P. Acute hepatitis B in a patient with antibodies to hepatitis B surface antigen who was receiving rituximab. N. Engl. J. Med. 2001 4;344:68–9.
2. Smolen JS, Keystone EC, Emery P, Breedveld FC, Betteridge N, Burmester GR, et al. Consensus statement on the use of rituximab in patients with rheumatoid arthritis. Ann. Rheum. Dis. 2007;66:143–50.
3. Artz AS, Somerfield MR, Feld JJ, Giusti AF, Kramer BS, Sabichi AL, et al. American Society of Clinical Oncology provisional clinical opinion: chronic hepatitis B virus infection screening in patients receiving cytotoxic chemotherapy for treatment of malignant diseases. J. Clin. Oncol. 2010 1;28:3199–202.

**1.5 Jak-inhibitors (VZV)**

1. Lee EB, Fleischmann R, Hall S, Wilkinson B, Bradley JD, Gruben D, et al. Tofacitinib versus methotrexate in rheumatoid arthritis. N. Engl. J. Med. 2014 19;370:2377–86.
2. Winthrop KL, Wouters AG, Choy EH, Soma K, Hodge JA, Nduaka CI, et al. The Safety and Immunogenicity of Live Zoster Vaccination in Patients With Rheumatoid Arthritis Before Starting Tofacitinib: A Randomized Phase II Trial. Arthritis Rheumatol. 2017 1;69:1969–77.
